# Accuracy and Precision of Visual Stimulus Timing in PsychoPy: No Timing Errors in Standard Usage

**DOI:** 10.1371/journal.pone.0112033

**Published:** 2014-11-03

**Authors:** Pablo Garaizar, Miguel A. Vadillo

**Affiliations:** 1 Deusto Institute of Technology, DeustoTech, Universidad de Deusto, Bilbao, Spain; 2 Department of Experimental Psychology, University College London, London, United Kingdom; 3 Primary Care and Public Health Sciences, King's College London, London, United Kingdom; National University of Singapore, Singapore

## Abstract

In a recent report published in PLoS ONE, we found that the performance of PsychoPy degraded with very short timing intervals, suggesting that it might not be perfectly suitable for experiments requiring the presentation of very brief stimuli. The present study aims to provide an updated performance assessment for the most recent version of PsychoPy (v1.80) under different hardware/software conditions. Overall, the results show that PsychoPy can achieve high levels of precision and accuracy in the presentation of brief visual stimuli. Although occasional timing errors were found in very demanding benchmarking tests, there is no reason to think that they can pose any problem for standard experiments developed by researchers.

During the last decades, computers have become an essential tool in psychological and neuroscientific research. Thanks to them, it is possible to present participants stimuli in different audiovisual formats and register different aspects of their reaction to those materials, including verbal judgments or response latencies. However, not all combinations of software and hardware are able to comply with the strict requirements of some experimental paradigms. For instance, researchers often need to present stimuli for very brief periods of time. Experiments on subliminal priming typically involve the presentation of words or images for intervals no longer than 16–50 ms [1], [2]. In these experiments, even small deviations from programmed durations (e.g., from 16 to 33 ms) can make a substantial difference in participants' ability to perceive stimuli. Similarly, some experimental paradigms require very accurate measurement of reaction times. Many of these experiments explore effects in the range of just 30–100 ms [3], [4]. Problems in the presentation of stimuli or in the logging of responses can affect the results of these kinds of experiments. Although measurement errors usually have a minimal impact on data when researchers average reaction times collected across many trials [5], [6], they can compromise more sophisticated analyses like, for example, fitting models to the distribution of reaction-times [7]–[9].

Fortunately for researchers, there is a wide variety of software packages available that have been carefully designed to comply with these strict requirements. In addition to proprietary software (e.g., E-Prime, Presentations), some outstanding open and free access alternatives are also available [10]–[13]. Among them, PsychoPy is quickly becoming a popular choice [14]. PsychoPy is a multiplatform software package for designing and conducting cognitive experiments that can run natively in Microsoft Windows, GNU/Linux and Apple Mac OS X. It is coded in Python, like many other alternatives available (e.g., Experiment Builder, PyEPL, OpenSesame, Vision Egg), and provides a graphical authoring tool (PsychoPy experiment builder) and a set of Python libraries for building experiments.

Unfortunately, in a recent report published in PLoS ONE, we found that the performance of PsychoPy degraded with very short timing intervals, suggesting that it might not be perfectly suitable for experiments requiring the presentation of very brief stimuli [15]. Although the performance of PsychoPy improved noticeably when running under a real-time operative system, important timing errors still remained for stimuli durations of 100 ms or less. However, there are reasons to suspect that the results of our previous tests might underestimate the potential accuracy of PsychoPy. Firstly, as noted by the author of PsychoPy himself [16], our study on the accuracy of PsychoPy was conducted with an early version of the software package that was almost 3 years old at the time the study was finally published. Our report ignored any improvements introduced in PsychoPy during that time. Secondly, the scripts used in our tests were generated using the experiment builder interface, which was not fully operative in that version. Furthermore, the experiment builder of the tested version did not allow defining stimulus durations in terms of ticks (i.e., display refreshes). Therefore, in our benchmark tests stimulus durations were defined in time units. This might have given rise to problems in the translation from the millisecond definition of stimuli to the corresponding number of ticks. Finally, given that the original study used a single computer for all tests, it is impossible to discard the possibility that the poor performance of PsychoPy reflected limitations of hardware, rather than genuine problems of software. The present study aims at providing an update of the performance of the more recent version of PsychoPy under ideal conditions.

## Methods

The main differences with respect to the previous study [15] are the version of PsychoPy being tested (1.80 instead of 1.64) and the specific scripts used to assess its accuracy. In the present study, the scripts were not created with PsychoPy's experiment builder. Instead, we adapted a benchmarking program developed by Jeremy Gray [17]. In addition, we have conducted our tests on updated operative systems.

### Methodology and Apparatus

Tests were conducted on two different computers: 1) Apple MacBook Pro 11,1 “Core i5” 2.4 13″ Late 2013 with 8 GB of RAM, a 13.3″ retina display (2560×1600 px), and an integrated Intel Iris 5100 graphics processor that shares memory with the system; and 2) Apple MacBook Pro 5,5 “Core 2 Duo” 2.26 13″ (SD/FW) Mid 2009 with 2 GB of RAM, a 13.3″ display (1280×800 px), and a NVIDIA GeForce 9400M graphics processor with 256 MB of DDR3 SDRAM shared with main memory. Three operative systems were installed on these machines: 1) MacOS X “Mavericks”; 2) Windows 7 64-bit Ultimate edition; y 3) Ubuntu Linux 13.10 “Saucy Salamander”. All tests were conducted in full screen mode, with the Bluetooth and the network connection (WiFi/Ethernet) disabled. The accuracy and precision of stimulus presentation was assessed using the Black Box Toolkit (BBTK), a set of photodetectors specifically designed to conduct benchmarking studies like the one reported here [18]. The BBTK detects changes in luminance from the photodetector and sends this information to the parallel port of an auxiliary computer, different from the one whose performance is being tested. This avoids any interference between the timing mechanisms used to generate the black to white and white to black transitions and the real-time application used to gather the data provided by the BBTK photodetector.

### Design and Procedure

For each combination of hardware and operative system we developed several full-screen animations with non-gradual, repeated white-black transitions. The duration of each keyframe was manipulated with values 1000, 500, 200, 100, 50, and 16.667 ms (60, 30, 12, 6, 3, and 1 display refreshes at 60 Hz, respectively), although, as explained below, not all durations were included in all tests. For each of these conditions, we collected data from 5 independent series of 60 seconds each. We limited our study to repeated white-black transitions because many studies about accuracy and precision in visual stimuli presentation use similar procedures [19]–[22] and because it is safe to assume that preparation times of this kind of simple stimuli will not affect their presentation times. Trying to measure the presentation times of complex or real-time generated stimuli from luminance changes usually gives rise to spurious errors that can be avoided by resorting to simple black-and-white transitions.

As mentioned above, to avoid any potential error in our PsychoPy code, we adapted a script previously published by Jeremy Gray, one of PsychoPy's developers, in a comment to our previous study [17]. We only modified the number of iterations in the trial loop (depending on the duration of each keyframe we needed more or less trials to complete the 60 seconds of measurement) and the number of durations of each experiments (we tested only one duration in each test, instead of 6). Apart from these two changes, the rest of the scripts were a verbatim copy of Gray's original.

## Results

Detailed data for all the tests reported below are available at the Open Science Framework public repository (https://osf.io/9dkgz/). The main goal of our tests was to find the threshold where PsychoPy started to show timing errors. For this reason, we did not test all stimulus durations (1000, 500, 200, 100, 50, and 16.667 ms) for all combinations of hardware and software. We started our analyses by testing the 1000, 500, 200, 100, and 50 ms conditions on MacOS X running on the MacBook Pro Late 2013. The results of these tests are shown in Table 1. As can be seen, the performance of PsychoPy was perfect for this range of stimulus durations. However, we did find timing errors when we proceeded to test the 16.667 ms interval. Upon further exploration of the benchmarking scripts, we found that the number of trials per loop was a key determinant of these timing errors. Specifically, we observed that errors were somewhat decreased when the number of trials per loop was reduced from 960 to 800. Following this observation, we further adjusted the number of trials per loop to 640 and observed that timing errors virtually disappeared under these conditions. These results have important consequences. Firstly, they confirm that PsychoPy is perfectly able to reach maximal precision even with the briefest stimulus presentation (16.667 ms). Secondly, they show that the number of trials per loop somehow affects the performance of PsychoPy. As a result, this parameter was also manipulated in the following tests.

**Table 1:** PsychoPy 1.80 timing tests on MacBook Pro Late 2013 under MacOS X “Mavericks”.

*Duration (ms)*	*Trials per loop*	*Test*	*Missed frames*
1000	60	1	0
	60	2	0
	60	3	0
	60	4	0
	60	5	0
500	120	1	0
	120	2	0
	120	3	0
	120	4	0
	120	5	0
200	300	1	0
	300	2	0
	300	3	0
	300	4	0
	300	5	0
100	600	1	0
	600	2	0
	600	3	0
	600	4	0
	600	5	0
50	1200	1	0
	1200	2	0
	1200	3	0
	1200	4	0
	1200	5	0
16.667	2400	1	1199
	2400	2	1198
	2400	3	1199
	2400	4	1200
	2400	5	1198
	2000	1	61
	2000	2	1173
	2000	3	1164
	2000	4	50
	2000	5	1173
	1600	1	0
	1600	2	1
	1600	3	0
	1600	4	0
	1600	5	0

We then explored the performance of PsychoPy under Windows 7 using the same computer. Table 2 shows the results of these tests. As can be seen, these tests yielded very poor results even for the less demanding conditions (1000 ms). This made us suspect that the timing errors observed in this condition could not be attributed to a problem in PsychoPy. Instead, these results are likely to be due to the deficient performance of the driver for the integrated Intel Iris 5100 graphics processor running on Windows 7, to the lack of precision and accuracy in Microsoft's latest operative systems [9], or to a combination of both. As shown below, PsychoPy shows a good performance under Windows 7 when a different graphics card and different drivers are used.

**Table 2:** PsychoPy 1.80 timing tests on MacBook Pro Late 2013 under Windows 7 64-bit Ultimate.

*Duration (ms)*	*Trials per loop*	*Test*	*Missed frames*
1000	60	1	130
	60	2	123
	60	3	123
	60	4	125
	60	5	124

We also tested PsychoPy on Ubuntu Linux running on the same computer. The results of these tests are summarized in Table 3. After checking that no timing errors were observed with 200 ms, we proceeded to test the 100 and the 50 ms conditions. Preliminary examination of the 50 ms interval did yield some timing errors. Therefore, we decided to adjust the number of trials per loop to 300. With this change, timing errors were no longer observed for the 50 ms interval. However, we did still observe timing errors in the 16.667 ms condition and we decided to further reduce the number of trials per loop to 150, which eliminated all timing errors in 4 out of the 5 tests conducted.

**Table 3:** PsychoPy 1.80 timing tests on MacBook Pro Late 2013 under Ubuntu Linux 13.10 “Saucy Salamander”.

*Duration (ms)*	*Trials per loop*	*Test*	*Missed frames*
200	300	1	0
	300	2	0
	300	3	0
	300	4	0
	300	5	0
100	600	1	0
	600	2	0
	600	3	0
	600	4	0
	600	5	0
50	300	1	0
	300	2	1
	300	3	0
	300	4	0
	300	5	0
16.667	300	1	15
	300	2	15
	300	3	2
	300	4	40
	300	5	26
	150	1	0
	150	2	15
	150	3	0
	150	4	0
	150	5	0

We took a similar approach to explore the performance of PsychoPy in the second computer, a MacBook Pro Mid 2009. Table 4 shows the results of the tests conducted with MacOS X running on this machine. In this case, we started by testing the 200 ms condition, which yielded no errors. We proceeded to conduct the tests in the 100 ms condition, where we did observe numerous timing errors. As in our previous tests, we followed up these tests changing the number of trials per loop from 1200 to 600. After this modification, timing errors were no longer observed in the 100 ms condition. Bearing this in mind, we tested the 50 ms condition with 600 trials per loop and we also found no timing errors. The same happened when testing the 16.667 ms interval. However, when we increased again the number of trials per loop in the 16.667 ms condition, we found again timing errors. This confirms that the timing errors that we found in PsychoPy so far should not be attributed to its ability to present very brief stimuli, but to the large number of trials per loop included in each test. Note that this large number of trials, although common for benchmarking studies, is rather unusual in the typical experiments designed by researchers.

**Table 4:** PsychoPy 1.80 timing tests on MacBook Pro Mid 2009 under MacOS X “Mavericks”.

*Duration (ms)*	*Trials per loop*	*Test*	*Missed frames*
100	600	1	0
	600	2	0
	600	3	0
	600	4	0
	600	5	0
50	1200	1	573
	1200	2	572
	1200	3	573
	1200	4	573
	1200	5	576
	600	1	0
	600	2	0
	600	3	0
	600	4	0
	600	5	0
16.667	1200	1	600
	1200	2	600
	1200	3	600
	1200	4	600
	1200	5	600
	600	1	0
	600	2	0
	600	3	0
	600	4	0
	600	5	0

The results obtained with Windows 7 running on the MacBook Pro Mid 2009 are shown in Table 5. In contrast with the results obtained with the MacBook Pro Late 2013, no timing errors were observed in the 100 ms condition. Isolated errors took place in the 50 ms condition, all of them conveniently reported in the PsychoPy log file. Surprisingly, only 1 out of the 5 tests conducted in the 16.667 ms condition yielded timing errors, even when the number of trials per loop was set to 1200. The outstanding performance of PsychoPy on Windows 7 even on adverse conditions is in stark contrast with its poor performance on the same operative system running on the MacBook Pro Late 2013. As explained above, everything suggests that these timing errors should not be attributed to a poor performance of PsychoPy. We found, however, that timing errors could still be observed if the number of trials per loop was set to 2400.

**Table 5:** PsychoPy 1.80 timing tests on MacBook Pro Mid 2009 under Windows 7 64-bit Ultimate.

*Duration (ms)*	*Trials per loop*	*Test*	*Missed frames*
100	600	1	0
	600	2	0
	600	3	0
	600	4	0
	600	5	0
50	1200	1	0
	1200	2	15
	1200	3	0
	1200	4	0
	1200	5	2
16.667	2400	1	600
	2400	2	600
	2400	3	600
	2400	4	600
	2400	5	599
	1200	1	0
	1200	2	0
	1200	3	1
	1200	4	0
	1200	5	0

Finally, Table 6 shows the results of the tests conducted with Ubuntu Linux running on the MacBook Pro Mid 2009. Before gathering these data, we found a problem in the execution of our tests: Preliminary tests showed that the stimulus durations registered by the BBTK photosensors doubled the expected values (e.g., white and black frames lasted 200 ms in the 100 ms condition). Surprisingly, this error was not reported in the PsychoPy log file. After commenting these results with the developers of PsychoPy, they informed us that in some configurations of Linux the graphics card is being told twice to wait for a vertical blank before proceeding, so every frame actually takes two frames. Because the frame time remains consistent, PsychoPy assumes that the frame rate of the monitor is 30 Hz (and not 60 Hz). Therefore, it does not report any missed frames (all frames look like the expected period by this measure). Fortunately, there was a simple solution. PsychoPy includes a property option to disable the wait for the next vertical blank (win.waitBanking = False). After implementing this change, we tested the 200 ms condition and found no timing errors. We also found no errors for the 100, 50, and 16.667 ms conditions when 600 trials per loop were requested. However, errors were found when the number of trials per loop was set to 1200.

**Table 6:** PsychoPy 1.80 timing tests on MacBook Pro Mid 2009 under Ubuntu Linux 13.10 “Saucy Salamander”.

*Duration (ms)*	*Trials per loop*	*Test*	*Missed frames*
200	600	1	0
	600	2	0
	600	3	0
	600	4	0
	600	5	0
100	1200	1	94
	1200	2	97
	1200	3	86
	1200	4	99
	1200	5	97
	600	1	0
	600	2	0
	600	3	0
	600	4	0
	600	5	0
50	1200	1	184
	1200	2	157
	1200	3	166
	1200	4	163
	1200	5	142
	600	1	0
	600	2	0
	600	3	0
	600	4	0
	600	5	0
16.667	1200	1	35
	1200	2	28
	1200	3	27
	1200	4	29
	1200	5	37
	600	1	0
	600	2	0
	600	3	0
	600	4	0
	600	5	0

## Discussion

When our previous study on the accuracy and precision of PsychoPy and other experimental software was originally published [15], the developers of PsychoPy [16] suggested that the timing errors that we detected could be due either to the fact that those tests were based on an earlier version of PsychoPy (1.64) or to the definition of stimulus durations in terms of time units instead of ticks (display refreshes). Actually, the latter problem was related to the former, given that the experiment builder of PsychoPy 1.64 did not allow defining durations in terms of ticks. It is very likely that the timing errors found in the previous study can be attributed to this feature of the testing procedure. Timing visual events based on timing intervals is known to be prone to artifacts, because those intervals often do not synchronize precisely with the hardware screen refresh interval, leading to uncertainties in the actual achieved display times.

In light of the present results it appears that an additional factor played a determinant role: The number of trials per loop implemented in each test. Although testing large numbers of trials per loop is common practise in software benchmarking, the parameters used in this kind of studies are rather unusual in psychological experiments. The divergence between the procedures used in cognitive research and the methods used in benchmarking has already been highlighted by Plant. As he mentioned in his comment to our original study, “flashing a bitmap over and over on idealised equipment is not representative of what real researchers do in the field! Their equipment is never ideal, their coding never as good as yours, their experiment is more complicated, they link to different equipment to yours… Or they are using a different version of the software to you” [23]. We might add that the divergence sometimes runs in the opposite direction: As the present study illustrates, sometimes the requirements of the software used to benchmark timing errors can be much more demanding than those of standard programs designed by experimenters. Given our results, a practical recommendation for cognitive researchers is that large numbers of trials per loop should be avoided by all means whenever it is possible.

The negative impact of this factor might be due to the large amount of information that PsychoPy has to log in relatively little time. Even though we disabled XLSX and CSV outputs, we still found errors with large numbers of trials per loop. Fortunately, this is more of a technical than a practical problem, because it only poses timing problems in highly unusual conditions. However, in light of the present results, it seems advisable to avoid complex data output formats, such as XLSX, when timing errors can be an issue, particularly for experimental programs requiring multiple loops.

It is also important to note that the performance of PsychoPy was also affected by details of the hardware and software used to run the experiment. Severe timing errors were observed in Windows 7 in one of the computers, possibly due to problems of the graphic card driver. Similarly, the configuration of the graphic card in Ubuntu Linux gave rise to unexpected timing errors that, fortunately, could be fixed using the appropriate property options in PsychoPy. These two examples illustrate that researchers can never take for granted that their software will reach the highest precision and accuracy levels under all circumstances. If a series of experiments demands compliance with strict timing requirements, the precision and accuracy of the experimental software should always be tested first.

Based on the results of our studies, we can offer some guidelines for researchers that are planning to use PsychoPy to conduct experiments with strict timing requirements. First, it is important to use suitable hardware equipment (i.e., a computer provided with a fast CPU, enough RAM, a dedicated graphics processor, and a display with low refresh rate) with the appropriate configuration (i.e., Bluetooth, Ethernet. Wi-Fi, Mobile and other kind of connections disabled; desktop visual effects disabled; antivirus, software updates, background programs, and other kind of asynchronous events sources disabled). Second, any configuration problem of the graphics processor should be detected and fixed (i.e., updating display's and graphics processor's drivers and using vendor's test utilities to benchmark them, if available). Third, it is advisable to use the last version of PsychoPy. It is free, and every update comes with new interesting features. Fourth, visual stimuli should be defined in durations in ticks (screen refreshes) and not in milliseconds. Fifth, it is preferable to avoid defining too many trials per loop in experiments. For experimental paradigms with large numbers of trials (i.e., experimental paradigms with several hundreds of trials, such as priming or contextual cueing [1], [3]), splitting the whole set of trials in several blocks is an easy way to avoid potential problems. Sixth, it is recommendable to analyse and reduce the impact of logging processes during the experiment (e.g., using XLSX log format is more demanding than TXT log format). In addition to these general recommendations, the precision and accuracy of the experimental setup should be tested prior to conducting the experiment. In most cases, PsychoPy's logging information should be enough to detect timing inaccuracies. In our study, all the timing errors except the one caused by the Nvidia graphics configuration in Linux were correctly reported by PsychoPy. To make sure that such faulty configuration is not being used unknowingly, researchers can define a human-measurable stimulus duration (e.g., 120 ticks = 2000 ms at 60 Hz) and check that the duration is not doubled (i.e., 4000 ms). If that is the case, there is a simple workaround in PsychoPy: Disabling waitBlank feature and defining stimuli durations in milliseconds and not in ticks (contrary to the previous recommendation).

To sum up, the present study shows that the most recent versions of PsychoPy can achieve the highest levels of precision and accuracy in the presentation of brief visual stimuli. There is no reason to think that occasional timing errors found in benchmarking tests with many trials per loop can pose any problem for standard experiments developed by researchers. Properly used, PsychoPy is an excellent tool for psychological research even under the most demanding conditions.
